# Molecules linked to Ras signaling as therapeutic targets in cardiac pathologies

**DOI:** 10.1186/s40659-021-00342-6

**Published:** 2021-08-03

**Authors:** Manuel Ramos-Kuri, Sri Harika Meka, Fabio Salamanca-Buentello, Roger J. Hajjar, Larissa Lipskaia, Elie R. Chemaly

**Affiliations:** 1 Instituto Nacional de Cancerología, Unidad de Investigación Biomédica en Cáncer, Secretarìa de Salud/Instituto de Investigaciones Biomédicas, Universidad Nacional Autónoma de México, Mexico City, México; 2 Researcher of the Facultad de Bioética, Cátedra de Infertilidad, Universidad Anáhuac, Mexico City, México; 3 Centro de Investigación en Bioética y Genética, Querétaro, México; 4 Division of Nephrology, Department of Medicine, Jacobs School of Medicine and Biomedical Sciences, State University of New York at Buffalo, Clinical and Translational Research Center, 875 Ellicott Street, Suite 8030B, Buffalo, NY 14203 USA; 5 University of Toronto Institute of Medical Science, Medical Sciences Building, 1 King’s College Circle, Room 2374, Toronto, ON M5S 1A8 Canada; 6 Phospholamban Foundation, Amsterdam, Netherlands; 7 INSERM U955 and Département de Physiologie, Hôpital Henri Mondor, FHU SENEC, AP-HP, and Université Paris-Est Créteil (UPEC), 94010 Créteil, France

**Keywords:** Ras-opathies, H-Ras gene, K-Ras gene, Ras pathway, Physiological hypertrophy, Pathological hypertrophy, MAP kinase, Calcineurin

## Abstract

**Abstract:**

The Ras family of small Guanosine Triphosphate (GTP)-binding proteins (G proteins) represents one of the main components of intracellular signal transduction required for normal cardiac growth, but is also critically involved in the development of cardiac hypertrophy and heart failure. The present review provides an update on the role of the H-, K- and N-Ras genes and their related pathways in cardiac diseases. We focus on cardiac hypertrophy and heart failure, where Ras has been studied the most. We also review other cardiac diseases, like genetic disorders related to Ras. The scope of the review extends from fundamental concepts to therapeutic applications. Although the three Ras genes have a nearly identical primary structure, there are important functional differences between them: H-Ras mainly regulates cardiomyocyte size, whereas K-Ras regulates cardiomyocyte proliferation. N-Ras is the least studied in cardiac cells and is less associated to cardiac defects. Clinically, oncogenic H-Ras causes Costello syndrome and facio-cutaneous-skeletal syndromes with hypertrophic cardiomyopathy and arrhythmias. On the other hand, oncogenic K-Ras and alterations of other genes of the Ras-Mitogen-Activated Protein Kinase (MAPK) pathway, like Raf, cause Noonan syndrome and cardio-facio-cutaneous syndromes characterized by cardiac hypertrophy and septal defects. We further review the modulation by Ras of key signaling pathways in the cardiomyocyte, including: (i) the classical Ras-Raf-MAPK pathway, which leads to a more physiological form of cardiac hypertrophy; as well as other pathways associated with pathological cardiac hypertrophy, like (ii) The SAPK (stress activated protein kinase) pathways p38 and JNK; and (iii) The alternative pathway Raf-Calcineurin-Nuclear Factor of Activated T cells (NFAT). Genetic alterations of Ras isoforms or of genes in the Ras-MAPK pathway result in Ras-opathies, conditions frequently associated with cardiac hypertrophy or septal defects among other cardiac diseases. Several studies underline the potential role of H- and K-Ras as a hinge between physiological and pathological cardiac hypertrophy, and as potential therapeutic targets in cardiac hypertrophy and failure.

**Graphic abstract:**

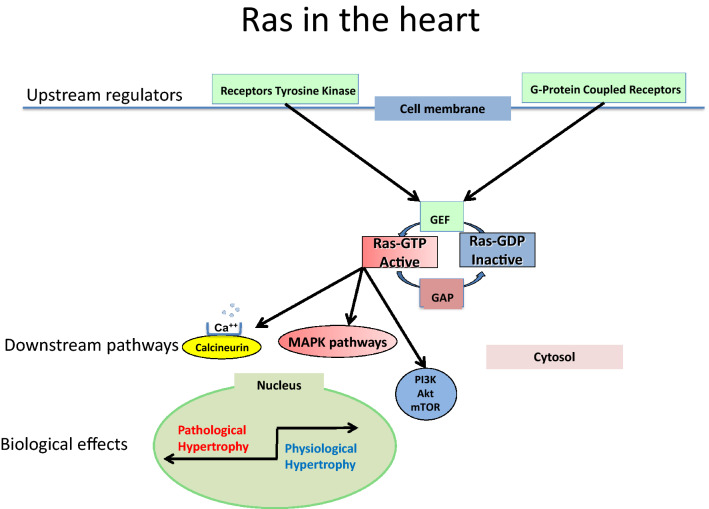

## Highlights

- The Ras (Rat Sarcoma) gene family is a group of small G proteins
- Ras is regulated by growth factors and neurohormones affecting cardiomyocyte growth and hypertrophy
- Ras directly affects cardiomyocyte physiological and pathological hypertrophy
- Genetic alterations of Ras and its pathways result in various cardiac phenotypes
- Ras and its pathway are differentially regulated in acquired heart disease
- Ras modulation is a promising therapeutic target in various cardiac conditions.

## Introduction

The Ras (Rat sarcoma) family of small G (Guanosine Triphosphate (GTP)-binding) proteins is composed of enzymes that hydrolyze GTP into GDP and represents a critical component of intracellular signal transduction [1]. Ras genes are ubiquitously expressed. Mostly known in cancer biology, these proteins are essential for cellular growth, differentiation and survival of many cell types [1, 2].

The Ras *superfamily* comprises more than a hundred cellular proteins, and is divided into five main *families*: Ras, Rho, Ran, Rab and Arf GTPases, depending on their genetic sequence, structure and function [3]. The Ras *family* is then divided into six *subfamilies*: Ras, Ral, Rit, Rap, Rheb, and Rad. The present review focuses on the role of the Ras *subfamily* in cardiac cells. There are three main human Ras genes, H-Ras, K-Ras and N-Ras. These genes are proto-oncogenes, and their mutated versions are responsible for neoplastic transformation; they are the most frequently mutated genes in all cancer types [3, 4]. In 1993, Thorburn et al. showed that H-Ras-Val12, an oncogenic mutant of H-Ras, induced hypertrophy of rat neonatal cardiomyocytes in vitro without oncogenic transformation [5]. The role of intracellular pathways involving small G proteins, including Ras, Rho and Rac, in cardiac hypertrophy is well established, as detailed in previous reviews [1, 2, 6–9]. The present review provides an update on the role of the Ras subfamily and its related pathways in cardiac diseases, mainly cardiac hypertrophy and heart failure (HF), where it has been widely studied, but also in genetic disorders of H- Ras, K-Ras, and N-Ras genes. The scope of this review extends from fundamental concepts to therapeutic applications.

## Molecular biology of the Ras subfamily

### Small G proteins

**Fig. 1:**
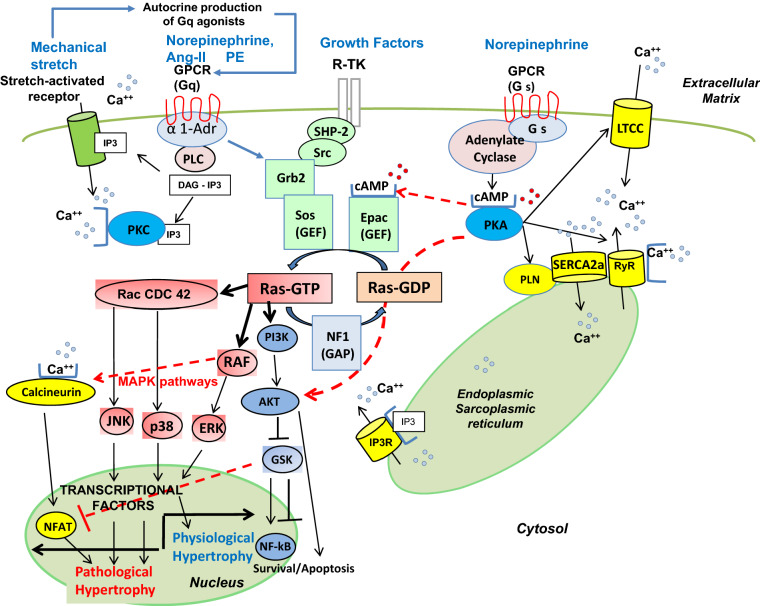
Ras and its related pathways. See text and references [1, 15, 21, 24, 44, 51, 52, 58, 60, 62] for details. (1) **Ras**: one of the central regulators of all the pathways shown. The activity of Ras is amplified by Guanine nucleotide exchange factors (GEFs) such as Sos and reduced by GTPase-activating proteins (GAP) such as Neurofibromin 1 (NF1). Ras is activated by various receptors at the cell membrane cell, particularly GPCR. (2) **Upstream activators of Ras**: **GPCR** Various stimuli activate GPCR, mainly Angiotensin II (AngII), through its type 1 receptor (Gq), and norepinephrine through its α1-adrenergic receptor (Gq) and β-adrenergic receptor (Gs). The subunit Gαs activates adenylate cyclase, which produces cyclic adenosine monophosphate (cAMP). **Gq** activates phospholipase C (PLC) which hydrolyzes phosphatidylinositol-4,5-bisphosphate (PIP_2_) to produce inositol-1,4,5-trisphosphate (IP_3_) and diacyl-glycerol (DAG). IP_3_ releases Ca^++^ from the sarco-endoplasmic reticulum through the IP_3_ receptor (IP_3_R). Ca^++^ and DAG activate protein kinase C (PKC). Ca^++^ activates calcineurine and calcineurine dephosphorylates and activates the cytosolic transcription factor NFAT-3 (Nuclear Factor of Activated T cells), inducing its nuclear translocation. Gq also activates Ras directly. **Gs** The action of the β-adrenergic receptor through Gs enhances cardiomyocyte contraction and relaxation. cAMP activates protein kinase A (PKA) which phosphorylates the L-type calcium channel (LTCC), facilitating voltage-gated Ca^++^ and entry and Ca^++^-induced Ca^++^ release from the sarco-endoplasmic reticulum through the Ryanondin receptor (RyR). RyR is also phosphorylated by PKA. PKA phosphorylates phospholamban (PLN) and lifts the inhibition on the sarco-endoplasmic reticulum Ca^++^ ATP-ase (SERCA), further enhancing cardiomyocyte function by facilitating Ca^++^ cycling. Ca^++^ can in turn bind to calcineurin. cAMP activates the GEF-Epac (exchange protein directly activated by cAMP). (3) **Downstream effectors of Ras**: **MAP-Kinases** Ras activates the Kinase pathways ERK, JNK and p38. The ERK pathway is related to physiological hypertrophy [25, 100, 109]. In contrast, the p38 and JNK, (known as SAPKs) pathways are activated by stress-related stimuli, and mediate pathological remodeling [62]. **Ca**^**++**^**/Calcineurin** This is the main pathway for pathological hypertrophy, also activated by Raf. **Akt pathway** Ras activates **Akt** through PI3K, with a dual cardio-protective effect, inhibition of apoptosis and pro-survival action, through NF-kB activation and NFAT inhibition [35]. However, NF-kB and GSK (Glycogen Synthase Kinase) can exert either pro- or anti-apoptotic effects. **Cross-talk points** are shown with a red arrow: (i) between Ras and the **Calcineurin** pathway through Raf [24, 25]. (ii) Between Adenylate cyclase and Ras pathways, with activation of Ras through Epac by cAMP. (iii) activation of ERK from PKA; and: (iv) Inhibition of NFAT by GSK

The role of small G proteins in signaling cardiac hypertrophy initiated by G-protein coupled receptors (GPCR) is well demonstrated [2]. Small G proteins act as a molecular switch, inactive in Guanosine Diphosphate (GDP)–bound state and active in Guanosine Triphosphate (GTP)–bound state, in which GTP induces a conformational change, subsequently activating downstream intracellular signaling pathways (Fig. 1) [1, 2, 7].

The spontaneous molecular switch activity of small G proteins is inefficient and is amplified by Guanine nucleotide exchange factors (GEFs) and reduced by GTPase-activating proteins (GAPs) (Fig. 1) [1, 2]. GEF activates Ras by exchanging GDP for GTP while GAP enhances GTP hydrolysis to GDP by accelerating the slow intrinsic GTPase activity of Ras; thus, GEF and GAP provide additional levels of modulation of the action of small G proteins [2]. Active Ras (GTP-bound) binds with high affinity to several molecular effectors through which it exerts its pleiotropic effects (Fig. 1) [10]. Signal transduction from the cell surface to the nucleus through Ras involves various and complex pathways, exerting diverse and even opposite effects such as cell proliferation or growth arrest, senescence or differentiation, apoptosis or survival [6, 10, 11]. The end-result of these pleiotropic effects of Ras depends on many factors such as cell type and gene expression pattern. For example, the Ras-related signaling pathways leading to cardiac hypertrophy and remodeling can be induced by α-adrenergic stimulation [12], among many other ligands (Fig. 1); and slight changes in the concentration of the α-adrenergic agonist phenylephrine (PE) can exert proliferative or anti-proliferative effects [13]. The role of Ras in transducing mechanical stimuli is more controversial [14, 15].

### The Ras gene subfamily in the heart

The three human Ras genes H-Ras, N-Ras, and K-Ras encode four isoforms of the Ras subfamily: H-Ras, N-Ras, and K-Ras4A and K-Ras4B, the latter two resulting from alternative splicing of the fourth coding exon 5 of the K-Ras gene [16]. To our knowledge, this alternative splicing of K-Ras has not been studied in cardiac cells.

Ras genes are very similar in primary protein structure but functionally different [17]. Their gene codes for a 189 amino acids protein, and the main difference between them is in the 25 amino acids at the carboxyl end. This carboxyl-terminal region is known as the hypervariable region of Ras, and is the essential signal for the localization of Ras isoforms to different micro-domains at the inner surface of the cellular membrane [18]. This hypervariable region bears very little identity between the Ras isoforms, as little as 15%, and contains the important CAAX box (C = cysteine, A = apolar amino acid and X = serine or methionine) in the carboxyl end of the protein, where the farnesyl lipid is attached [18, 19].

## Physiological and pathological cardiac hypertrophy

Cardiac hypertrophy is a heterogeneous set of phenomena [2, 20]. It includes physiological hypertrophy resulting from normal cardiac growth or a response to aerobic exercise and pregnancy [21]. Physiological hypertrophy is adaptive and enhances cardiac structure and function [2, 9, 20]. On the other hand, pathological hypertrophy, which may be initially adaptive and subsequently maladaptive, occurs in response to stressors such as hypertension, excess mechanical loading caused by structural lesions, or mutations in genes encoding sarcomeric proteins. Pathological hypertrophy progresses towards HF, with fibrosis and arrhythmias, when sustained [2].

When faced with a pro-hypertrophic stimulus, the myocardium remodels structurally and molecularly within a spectrum of responses between the extremes of either physiological or pathological hypertrophy [21, 22]. In a comparative study of pressure and volume overload left ventricular hypertrophy and failure, we have demonstrated an association of the propensity for ischemia to myocardial fibrosis, suggesting that oxygen supply–demand imbalance may drive the myocardium to pathological hypertrophy [23].

## Role of H-, K- and N Ras in cardiac hypertrophy

### H-Ras in cardiac hypertrophy

As previously mentioned, pioneering studies of oncogenes in cardiomyocytes in vitro showed that oncogenic H-Ras induced hypertrophy [5]. In contrast to the effects of such mutants on other cell lineages, they did not activate malignant transformation of cardiomyocytes, but instead induced cardiac hypertrophy the with expression of classical markers of pathological hypertrophy such as Atrial Natriuretic Factor (ANF), Skeletal Muscle α Actin (SkM-α Actin), β-Myosin Heavy Chain (β-MHC) and c-Fos in neonatal cardiomyocytes in vitro [5, 14, 15].

The central role of H-Ras in pathological cadiac hypertrophy resides in the activation of several pathways, such as the transcriptional effects of the activation of the Ras/Raf/MEK/MAPK pathway (see “Abbreviations”—MAPK, Mitogen-Activated Protein Kinase and MEK, MAPKK (MAP kinase kinase) that activates a MAPK, extracellular signal-regulated protein kinase (ERK), or the Calcineurin/Nuclear Factor of Activated T cells (NFAT) pathway [1]. These pathways lead to a decrease in the expression of the sarcoplasmic reticulum calcium ATPase 2a (SERCA2a), thus promoting pathological cardiomyocyte hypertrophy [1].

The normal isoform of H-Ras stimulates a physiological form of cardiac hypertrophy, through the ERK pathway, while its oncogenic mutant H-Ras-Val12 (constitutive active) stimulates pathological hypertrophy through the Calcineurine/NFAT pathway [24, 25]. Also, the mechanism and pattern of phosphorylation or auto-phosphorylation of ERK1/2 can lead to either physiological or pathological cardiac hypertrophy [20, 26].

#### H-Ras and the cardiomyocyte structure

The effect of Ras-Val12 on the sarcomeric structure of neonatal cardiomyocytes in vitro has been controversial, since two independent studies reported that Ras-Val12 induced hypertrophy with enhanced or unchanged sarcomeric organization [5, 27], while other in vitro studies showed that Ras-Val12 caused myofibrillar disorganization [25], or reduced myofibrillar density [28]. Also, in transgenic mice, the active mutant H-Ras-Val12 caused obstructive hypertrophy with myofibrillar disarray, similar to hypertrophic cardiomyopathy (HCM) [29, 30], or severe hypertrophy with diastolic dysfunction [27, 30]. In line with this, Zheng et al. found that transgenic mice overexpressing H-Ras-Val12 under control of an α-MHC promoter, developed postnatal and lethal HF [27]. At the cellular level, H-Ras-Val12 induced several HF-related phenomena: i) it reduced the L-type Ca^++^ channel current, and sarcoplasmic reticulum Ca^++^ uptake [25]; ii) it reduced the expression of SERCA2a and the phosphorylation of phospholamban, leading to diastolic dysfunction [27].

Furthermore, the expression of H-Ras is increased in the myocardium of patients with genetic HCM secondary to β-myosin heavy chain or troponin T mutations [31]. Importantly, the hypertrophic process initiated by Ras-Val12 can be reversed: suppression of an inducible promoter on H-Ras-Val12 reverses the associated cardiomyopathy [32].

### Cardiac role of K-Ras

Relatively few studies have investigated K-Ras in the heart, but it is clearly linked to cardiac cell proliferation. The first observation in this way was in the K-Ras^−/−^ knock-out mice, which die on embryonic day 15.5 with extremely thin left ventricular walls [33]. The proliferative role of K-Ras in cardiac cells was corroborated in mice with active mutant K-RasV14I, which causes cardiac enlargement secondary to cardiomyocyte hyperplasia (increased number of cells) without cardiomyocyte hypertrophy [34]. This proliferative role of K-Ras is also observed in other tissues: mutations of K-Ras are associated with 25–30% of all cancers, versus 3% for H-Ras and 8% for N-Ras [4]. Interestingly, when K-Ras is only partially down-regulated in heterozygous K-Ras^±^ mice, the heart is normal at birth, with even improved structure and function under pressure overload [35].

The underliying mechanism of this differential function between H-Ras (hypertrophy) and K-Ras (proliferation) in cardiac cells is not known, to our knowledge. In colorectal cancer cells, for example, K-Ras sustains cell proliferation through p38, even when MEK1/2 and ERK1/2 are inhibited with inhibitory RNAi [36].

### N-Ras and the heart

Among genes of the Ras subfamily, N-Ras appears to have the least functional importance in the heart. The cardiac role of N-Ras is not yet clearly determined, although active mutations of N-Ras cause Noonan syndrome, or Ras-opathies, but much less frequently than H- or K-Ras mutations [37]. Patients with Noonan syndrome and N-Ras mutations also present congenital heart defects like HCM or pulmonary stenosis [37].

### Differences and similitudes between Ras isoforms

#### Microdomain localization

Ras isoforms have a very similar protein structure. Functional differences between Ras isoforms are due, at least in part, to their localizations at different domains of the plasma membrane. H-Ras is equally localized in caveolae, lipid rafts and disorganized membrane. K-Ras4A is localized preferentially in disorganized nonraft plasma membrane. K-Ras4B is directly shuttled to the plasma membrane, thanks to a specific polybasic lysine-rich sequence at its carboxyl-terminal region [38, 39]. Finally, N-Ras localizes to caveolin-positive and caveolin-negative domains [18, 39]. However, to our knowledge, these subcellular localization differences have not been studied in cardiac cells.

#### Compensatory functions between Ras isoforms

The functions of the three genes of the Ras subfamily in the heart are mutually compensatory, as shown in a few examples. H-Ras^−/−^ and N-Ras^−/−^ knock-out mice have a normal phenotype; even mice with a double knock-out, H-Ras^−/−^ plus N-Ras^−/−^, have a normal phenotype including a normal heart [40, 41]. This compensatory function between Ras genes is further supported by evidence from a knock-in model, in which the H-Ras coding sequence replaces the K-Ras^−/−^ locus; even in a triple knock-out mouse (H-Ras^−/−^, K-Ras^−/−^ and N-Ras^−/−^), the knock-in H-Ras supplies the Ras function resulting in normal embryonic development [41, 42]. However, the adult mice of this H-Ras knock-in model developed a dilated cardiomyopathy due to high systolic and diastolic blood pressure, suggesting that K-Ras is the only absolutely required for normal cardiovascular function [41, 42].

Despite these similitudes and complementarities between them, Ras isoforms are not functionally redundant [18, 38], as illustrated by the different cardiac phenotypes seen in Ras-opathies, depending on the affected Ras isoform, as reviewed and described elsewhere [1, 37, 43].

## Ras-related network in cardiomyocytes

The Ras signaling network connects signals from several extracellular receptors to intracellular signaling pathways and, in turn, activates multiple downstream effectors, resulting in a variety of metabolic effects.

Extracellular receptors linked to the Ras network include various tyrosine-kinase receptors ((Epidermal Growth Factor (EGF), Platelet-Derived Growth Factor (PDGF), among others) and GPCRs (Fig. 1).

### Receptor tyrosine kinases (RTKs)

Various polypeptidic growth factors, cytokines and hormones bind to the extracellular domain of a RTK, activating the RTK cytoplasmic kinase domains. This allows a tyrosine in the cytoplasmic portion of each receptor monomer to be *trans*-phosphorylated by its partner receptor, leading to activation of downstream signal transduction pathways, such as the Ras/MAPK signaling cascade.

### Ras adaptor proteins

RTK activate Ras through adaptor proteins including SHP-2, Src, and Grb2 [1, 44] (Fig. 1). Importantly, more than 50% of patients with Noonan syndrome display a pathogenic mutation in the PTPN11 gene encoding SHP-2 protein [7]. In line with this, Thorburn et al. [5] reported that, in cardiomyocytes, the active mutant of Src-F527 activated H-Ras and induced cardiac hypertrophy.

### Positive and negative regulation of Ras

As mentioned earlier, the activity of Ras is positively regulated through GEFs. Examples of GEFs include Son of sevenless (Sos) and the Exchange Protein directly Activated by cyclic adenosine monophosphate (cAMP) (Epac). Mutations of the GEF Sos are associated with Noonan Syndrome [1]. Ras is negatively regulated through GAPs, like neurofibromin 1 (NF1) [2]. GAPs terminate Ras signaling by binding to the activated G proteins to stimulate their GTPase activity. The two most studied GAPs in the Ras pathway are NF1 and Carabin [45–49]. Total NF1 deletion in mice results in hyper-proliferation of the cardiac outflow tract, with redundant tissue at the endocardial cushions [47]. Mice with specific loss of NF1 in the heart developed marked cardiac hypertrophy in adult life, with progressive dilated cardiomyopathy and fibrosis [49]. Similarly, total deletion of carabin in mice promotes the development of major cardiac hypertrophy in a pressure overload model [45].

### G-Protein Coupled Receptors (GPCRs)

There are two major signaling transduction pathways involving the GPCRs: the cAMP signaling pathway (Gαs –coupled receptors) and the phosphatidylinositol signaling pathway (Gαq/_11_ –coupled receptors).

#### cAMP signaling pathway

In cardiomyocytes, the cAMP signaling pathway is coupled to the activation of the β-adrenergic receptors (β-AR) by catecholamines (Fig. 1). Within the cardiomyocyte, cAMP activates the cAMP-dependent protein kinase A (PKA), inducing the phosphorylation of the L-type Ca^++^ channels (LTCCs), the ryanodine receptors/channels (RyRs), and of the negative regulator of SERCA2a phospholamban (PLN), thus amplifying the intracellular calcium transient and mediating strong inotropic, lusitropic and chronotropic responses [50]. The cAMP pathway also activates the exchange protein Epac1 linked to H-Ras signaling (Fig. 1) [1, 51, 52]. Epac1 is upregulated in mouse models of isoproterenol-induced left ventricular hypertrophy, and in models of pressure overload-induced hypertrophy and HF, indicating an important role for Epac1 in pathological cardiac remodeling [53]. The pro-hypertrophic signaling resulting from the stimulation of the β1-AR activates the β-arrestin-Epac1 signaling complex including two small GTPases, Rap2 and Ras, as well as the Ca^++^/calmodulin-dependent protein Kinase II (CaMKII). Upon β_1_-AR stimulation, Epac1 activates Ras, Calcineurin, and CaMKII, rather than its classical effector Rap1, leading to cardiac hypertrophy [54]. A dominant negative form of Ras, RasS17N, decreased the effect of Epac1-induced cardiac hypertrophy, highlighting the role of Ras, rather than Rap1, in the β-AR /cAMP/Epac hypertrophic signaling pathway [54].

#### Phosphatidylinositol signaling pathway

In cardiomyocytes, the G_αq/11_ pathway is mediated by several membrane receptors, including the α1-adrenergic receptor (α1-AR) activated by catecholamines and the angiotensin-II (AngII) receptor type 1 (AT1R). The effector of the G_αq/11_ pathway is phospholipase C (PLC), which catalyzes the hydrolysis of membrane-bound phosphatidylinositol 4,5-bisphosphate (PIP_2_) into the second messengers inositol 1,4,5 trisphosphate (IP_3_) and diacylglycerol (DAG), both of which can affect directly or indirectly plasma membrane or sarcoplasmic Ca^++^ channels such as the IP_3_ receptor (IP_3_R), resulting in sustained increase in cytosolic Ca^++^. DAG and cytosolic Ca^++^ contribute to activation of protein kinase C (PKC). Elevated intracellular Ca^++^ also activates the prohypertrophic Ca^++^/calmodulin signaling pathway [55–57]. In cardiomyocytes, catecholamines also activate the α1-AR, inducing a long-term hypertrophic response mediated by Ras-GTP/MAPK and Ras-GTP/JNK (JNK, Jun NH_2_-terminal kinase, see “Abbreviations”) pathways [6, 11, 58] (Fig. 1).

AngII was also shown to activate directly the Ras-Raf-MAPK pathway, as well as two other MAPK pathways, JNK and p38 [59]. Indeed, inhibition of AT1R by candesartan abrogates the Ras-Raf-MAPK pathway, and partially downregulates the other two MAPK pathways, JNK and p38 [59, 60]. Antagonizing the actions of AngII is an established therapeutic strategy in patients with cardiac hypertrophy and HF [21, 61].

### Signaling pathways downstream of Ras

Ras modulates at least nine downstream signaling pathways; mostly involving MAPK[58]. Three kinase pathways are named after their final kinase: ERK, JNK, and p38 (Fig. 1). Ras also modulates the Akt/mTOR –signaling network and some non-kinase pathways, including the Ca^++^/Calcineurin pathway [58]. Ras regulates physiological cardiomyocyte growth, mainly through the Ras/Raf/ERK pathway, and pathological cardiac remodeling through other kinase cascades: JNK, p38 and ERK5, as well as the Ca^++^ signaling pathway [6, 11, 22, 25, 58] (Fig. 1).

#### Ras-MAPK pathways

Ras is linked to MAPK pathways through Raf, as part of the of the classical Ras/Raf/MEK/ERK1/2 pathway [62].

Exlusive activation of the ERK signaling pathway in cardiomyocytes mediates physiological hypertrophy. Transgenic mice with an activated MEK1 gene with cardiac-restricted expression displayed a physiological form of cardiac hypertrophy and a partial resistance to cardiomyocyte apoptosis [48]. Similarly, expression of dominant negative Raf (DN-Raf-1) in mice has no effect on cardiac function at baseline, but promotes cardiomyocyte apoptosis and increases mortality in the setting of pressure overload [63]. Finally, total ablation of c-Raf-1 in mouse hearts led to dilated cardiomyopathy, even in the absence of external stress [64].

JNK and p38 signaling pathways are also known as the Stress Activated Protein Kinases (SAPK) pathways. These pathways respond to several stressful stimuli like cytokines, the Tumor Necrosis Factor (TNF) and interleukin 1, or to physical stimuli like osmolar or oxidative stress, ionizing radiation, or mechanical hypertrophic stimuli, like cellular stretching [8]. Hyperactivation of SAPK plays a critical role in the development of pathological cardiac hypertrophy and HF [48]. Ras can activate the SAPK pathways by Rac1, a small GTPase of the Ras superfamily or by PI3K/AKT/mTOR network.

The role of the Ras/JNK signaling pathway in hypertrophic cardiac remodeling was established in transgenic mice expressing the oncogenic H-Ras-Leu61 mutant [12]. Other studies demonstrated that inhibition of JNK kinase attenuated cardiac hypertrophy in animal models [60, 65].

The p38 signalling pathway was shown to play a role in apoptosis and hypertrophic remodeling of cardiomyocytes [66]. Several studies pointed to the direct link between Ras and p38 kinases. Indeed, stimulation of rat neonatal cardiomyocytes with PE, AngII or Endothelin-I activates p38, JNK and ERK [21].

Furtrhermore, dominant negative Ras and the Ras inhibitor manumycin completely inhibited AngII –induced ERK activation [67]. AngII induces Ras/ERK activation partially via the Ca^++^/calmodulin-activated tyrosine kinase PYK2 (proline-rich tyrosine kinase 2), which is involved in Ca^++^-dependent tyrosine phosphorylation of ERK, and in the binding of GTP to p21^Ras^, demonstrating a cross-talk between Ca^++^ and tyrosine-kinase signalling pathways [67]. PYK2 activation plays a critical role in the induction of pressure overload-induced cardiomyocyte hypertrophy [68]. Given the fact that Ca^++^ overload and Ca^++−^activated signaling pathways are also critically involved in pathological cardiomyocyte remodeling, PYK2 may be a key regulator of at least some signaling pathways leading to the induction of cardiac hypertrophy and its progression to HF [68].

#### Ca^++^/Calcineurin/NFAT pathway

**Fig. 2:**
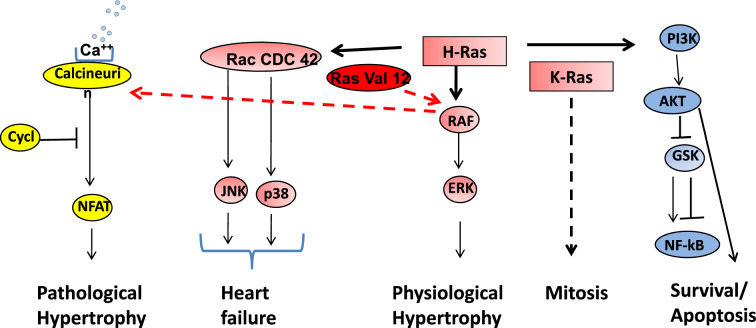
Summary of key downstream Ras pathways in the cardiomyocyte and their modulators. Cyclosporine (Cycl) inhibits calcineurin and cardiac hypertrophy, but causes HF. Ras-Val12 activates pathological cardiac hypertrophy through calcineurin, activated by Raf. K-Ras activates cardiomyocyte mitosis, although the specific pathways utilized by K-Ras are not studied (see text for references)

The Ca^++^/calcineurin signaling pathway plays a central role in pathological cardiac remodeling [57, 62]. Increases in resting intracellular Ca^++^ concentration in cardiomyocytes activates the Ca^++^/calmodulin regulated phosphatase 2B (calcineurin). Calcineurin dephosphorylates and activates the cytosolic transcription factor NFAT-3, inducing its nuclear translocation [57, 62] (Fig. 1). NFAT is involved in the induction of genes related to hypertrophic cardiomyocyte remodeling, leading to cardiac dysfunction and HF [57, 62] (Fig. 2). The role of Ras in activating the Ca^++^/calcineurine signaling pathway was demonstrated in animal models with cardiac overexpression of oncogenic active H-Ras [69]. Active mutants of Raf also activate Calcineurin, making Raf a cross-talk point between the Ras/Raf/ERK and the Ca^++^/Calcineurin pathways [24]. In line with this, several gain-of function mutations of Raf are associated with activation of Ca^++^/calcineurine signaling and pathological cardiac hypertrophy, as in Noonan and LEOPARD syndromes [1, 24].

#### PI3K/Akt/mTOR signaling network, another important Ras-related pathway

The PI3K/Akt/mTOR signaling network is a well-known player in cell metabolism, survival and growth [26, 70]. Even though Ras is not referred as a member of the mTOR signaling network, numerous studies, including studies from the Proud laboratory, have established the links between Ras/ERK, Ras/Akt and mTORC1 dependent protein synthesis [35, 58, 66, 71–73] (Fig. 1). Indeed, the Proud group has established that the activation of protein synthesis by the α1-AR agonist PE and by endothelin-1 is blocked by the mTORC1 inhibitor rapamycin, indicating that PE and endothelin-1 activate mTORC1 via Ras/MEK/ERK signaling in cardiomyocytes [71, 72]. Therefore, the expression of constitutively active MEK1 caused activation of protein synthesis in a rapamycin-dependent manner [71, 72]. Another study from this group reported that overexpression of the Ras homolog Rheb in rat cardiomyocytes was sufficient to activate mTOR1 and induced cardiomyocyte growth, but not the expression of markers of pathological remodeling ANF and B-type natriuretic peptide [73], suggesting that exclusive activation of mTORC1 promotes a rather physiological than pathological hypertrophy [73]. The mechanism of the interaction of mTORC1 and Ras/MEK/ERK is not completely understood, but might be related to the inhibition of Akt downstream effector proteins TSC1 and TSC2. The TSC1/2 complex acts as a GTPase –activator protein for the small GTPase Rheb, and Rheb GTP activates mTORC1 signaling [74]. Other effectors regulated by Akt include caspases, glycogen synthase kinase-3 (GSK3) and NF-kB [8, 26, 48, 70]. PI3K/Akt/mTOR is a complex network with several feedback loops, each mediating separate cellular processes, thus, the consequences of experimental Akt activation on cardiac function were variable, depending on experimental conditions. For example, in patients with HF, Akt activation is cardioprotective [75]. Akt was also shown to promote physiological cardiac hypertrophy and, under prolonged stimulation, pathological hypertrophy and HF [34, 76, 77]. Activation of GSK and NF-kB also enhances cardiac cell survival by its anti-apoptotic effect (Fig. 2) [34, 58], but can also mediate cardiac hypertrophy [34, 78]. These studies highlight interconnections at several levels between Ras/MEK/ERK and PI3K/Akt/mTOR signaling networtks controling protein synthesis, cardiomyocyte growth and remodeling.

## Ras and other cardiac diseases

### Ras-opathies and the heart

Several related syndromes known as Ras-opahties are due to germline mutations in genes of the Ras subfamily or in the Ras/Raf/ERK pathway (Fig. 1 and Table 1. These entities involve mutations in more than 20 genes [43], and manifest as heart diseases [79]. Jhang et al. analyzed a series of 155 patients with Ras-opahties, 118 of them had a cardiac anomaly, most commonly pulmonary stenosis, followed by atrial septal defect and HCM [79].

**Table 1:** Summary of H, K and N-Ras mutants with gain or loss of function, in vitro (cardiomyocytes in culture) and in vivo (rodent models and clinical syndromes)

	Increased function	Decreased function
H-Ras	**In vitro**: Ras Val12 [25, 28]: Hypertrophy with sarcomeric disruption Inhibition of cardiomyocyte beating **In vivo**: Ras Val12 in mice [12, 41]: Hypertrophic cardiomyopathy H-Ras wild type compensates K-Ras^−/−^ in mice **Clinical**: Costello syndrome [81] Hypertrophic cardiomyopathy Arrhythmias	**In vitro**: DN-Ras [25]: Enhancement of cardiomyocyte beating Enhancement of sarcomeric structure **In vivo**: experiments of H-Ras inhibition [25, 33]: DN-Ras favors physiological cardiac hypertrophy in a rat model of pressure overload Potential therapy for pathological cardiac hypertrophy and heart failure H-Ras^−/−^ mice: normal heart and offsprings **Clinical**: no data
K-Ras	**In vitro**: Active K-Ras V14I in cardiomyocyte [34]: Increased mitosis **In vivo**: K-Ras V14I mice [34]: Hyperplasia with no hypertrophy **Clinical**: Noonan or cardio-facio-cutaneous syndromes [80]: Hypertrophic cardiomyopathy Atrial and ventricular septal defects Pulmonary valve stenosis	**In vitro**: No data **In vivo**: K-Ras^−/−^ mice [33, 35]: Very low proliferation of cardiomyocytes, lethal at embryonic day 15.5 with extremely thin ventricular walls K-Ras is the only essential gene in the Ras subfamily K-Ras ^±^ mice: normal at birth and protected in pressure overload **Clinical**: no data
N-Ras	**In vitro**: No data on cardiac cells **In vivo**: N-Ras G12D mice [110] Mutated mouse embryos with cardiac malformations mimicking Noonan Syndrome: ventricular septal defects, double outlet right ventricle, hypertrabeculated/thin myocardium and pulmonic valve stenosis **Clinical**: Noonan syndrome—some cases [37]	**In vitro**: No data **In vivo**: N-Ras^−−/−^ mice [33]: Normal heart, normal offsprings **Clinical**: no data

Most data on N-Ras come from clinical reports. There are no clinical reports of decreased function of H-, K- or N-Ras

Noonan syndrome was the first Ras-opathy reported [43], is the second most common syndromic cause of congenital heart disease [80], and is associated with mutated genes in the Ras signaling pathway: SHP2 (PTPN11), SOS1, K-Ras or C-RAF among others. As previously stated, mutations in the PTPN11 gene are present in more than 50% of patients with Noonan syndrome [43]. On the other hand, LEOPARD syndrome has been re-classified as Noonan syndrome with lentigines, and is caused by two of the genes causing Noonan syndrome, SHP-2 or B-RAF [43]. At the cardiac level, 66% of Noonan syndrome patients presented with pulmonary valve stenosis and 14% with HCM [80, 81].

There is also a significant clinical overlap between cardio-facio-cutaneous (CFC) and Costello syndromes [82]. The CFC syndromes are genetically heterogeneous, with mutated genes downstream of Ras in the Ras-MAPK pathway: K-Ras, BRAF, MAPK1 and MAPK2, BRAF being the most frequently mutated gene [82]. In contrast, Costello syndrome is almost exclusively associated with H-Ras mutations in codons 12 or 13 [82]. Only 44% of patients with Costello syndrome have congenital heart defects, with valvar pulmonary stenosis, HCM, or atrial tachycardia. Costello syndrome is also associated with a higher risk of malignancies like rhabdomyosarcoma or bladder cancer [83].

Mutations of Ras negative regulator NF1 are associated with characteristic neurofibromas (Schwann cells neoplasms) and cardiac hypertrophy [84]. However, the cardiac phenotype is only present among patients with large NF-1 deletions: a clinical study found that only 6 out of 16 patients with neurofibromatosis had congenital heart disease, and only three of them had cardiac hypertrophy [84].

### Ras and heart failure

HF is a leading cause of sudden death, frequently preceded by pathological cardiac hypertrophy.

Haq et al. compared the activity of five major signaling pathways related to Ras, in hearts of patients with cardiac hypertrophy (secondary to hypertension alone or hypertension and coronary artery disease), versus patients with HF [75]. In that study, kinases pathways (MAPK, p38 and SAPK) showed a low level of activation in hypertrophy, but were highly activated in HF. In contrast, the calcineurin pathway was highly activated in hypertrophy, but to a lesser extent in HF. Finally, the Akt pathway was not active in cardiac hypertrophy, but highly active in HF [75].

### Ras and cardiac arrhythmias

Hypertension is the most common and modifiable risk factor for the development of atrial fibrillation [85, 86]. Activation of the renin–angiotensin–aldosterone system in hypertension leads to left atrial enlargement with secondary fibrosis and conduction abnormalities, resulting in atrial fibrillation [87]. A study of hypertensive rats showed that telmisartan was more effective in reducing the arrhythmogenic potential than valsartan at similar blood pressure control. Hypertension activates the Ras-ERK pathway and inhibits the PI3K-Akt-endothelial nitric oxide synthase (eNOS) pathway, causing structural remodeling and atrial arrhythmias. Telmisartan suppressed Ras-ERK signalling and activated PI3K-Akt-eNOS to a greater extent than valsartan [88]. This in turn led to significantly decreased myocyte size, intersitial fibrosis, apoptotic index and shortened duration of atrial arrhythmia when induced in Telmisartan-treated, spontaneous hypertensive rats [88]. Other studies have shown that the atrial expression ERK1/2 is increased in patients with atrial fibrillation compared with those in sinus rhythm [89].

### Ischemic preconditionning and Ras

Two different experiments have shown that Ras inhibition had a beneficial effect in ischemic preconditioning of the myocardium: one with the farnesyl transferase inhibitor FPT-III [90], and the other one with lovastatin [91]. However, in both cases, the protective effect was observed only when the Ras inhibitor was administered prior to the ischemic event [90, 91]. When FPT-III was given during re-perfusion, the degree of improvement in left ventricular contractility was significantly less [90]. Lovastatin achieved this effect at the very high dose of 15 mg/kg, close to 100 times higher than the doses used to treat clinical hyper-cholesterolemia.

## Modulation of Ras as a possible therapy for cardiac hypertrophy and HF

A large variety of pharmacological and non-pharmacological therapies are available in HF [61], nonetheless, there remain unmet therapeutic needs for HF as a common and serious condition [92, 93]. The results of cellular and animal studies have highlighted the central role of Ras in the control of numerous signaling pathways involved in physilogical and pathological cardiac remodeling. Thus, molecules linked to Ras signaling are potential therapeutic targets in cardiac pathologies.

### Molecular strategies

The most specific modulator of H-Ras is its dominant negative mutant DN-RasN17, but its clinical use is highly challenging. It attenuates pathological ventricular remodeling (versus physiological) in a rat model of pressure overload hypertrophy, as attested by the decreased expression of pathologic cardiac hypertrophy markers ANF and β-MHC, improved sarcomeric function and enhanced Ca^++^ transient in cardiomyocytes [25]. In spontaneously hypertensive rats, a traditional Chinese medicine suppresses left ventricular hypertrophy through down-regulation of Ras and ERK1/2 expression [94].

### Modulation of Ras prenylation

Addition of a farnesyl molecule to the aminoacid sequence CAAX in the carboxyl end of the Ras protein is a post-translational modification needed to localize Ras at the cell membrane. This prenylation of Ras can be inhibited by statins or by inhibitors of the Farnesyl Transferase, which makes these molecules Ras modulators.

#### Statins: Inhibition of farnesyl synthesis

Statins are a group of anti-hypercholesterolemic drugs that inhibit the syntesis of the farnesyl molecule, and have been used to inhibit Ras prenylation and subsequently cardiac hypertrophy in animal models. More precisely, statins inhibit the hydroxymethyl-glutaryl-conezyme A (HMG-CoA) reductase, which synthetizes isopentyl, an important precursor of farnesyl (Table 2) [95]. In human endothelial cells, lovastatin inhibited Ras, Rho, and Rap prenylation [95]. Indolfi et al. used simvastatin, inhibiting Ras and preventing the development of left ventricular hypertrophy in rats with aortic banding [96]. However, both experiments needed a very high concentration of statins, a thousand and a hundred times higher respectively, than the dose used in hypercholesterolemia [95, 96]. Finally, out of three clinical trials of Ras inhibition with lovastatin in patients with neurological deficits secondary to Ras-opathies, two suggested a modest benefit [97, 98], while a third did not find any significant effect after 16 weeks of treatment [99].

**Table 2:** Summary of Ras and Ras pathways modulators in the heart

Group	Molecule	Mechanism of action	References
Inhibitors of Angiotensin-II	Candesartan	Angiotensin-II AT1R receptor antagonist	Established therapy of clinical cardiac hypertrophy and HF
	Captopril	Angiotensin-converting enzyme (ACE) inhibitor	Established therapy of clinical cardiac hypertrophy and HF
Inhibitors of Ras farnesylation	Statins	Inhibits synthesis of farnesyl by inhibition of HMG-CoA reductase	Lipid lowering therapy with broad clinical applications in cardiovascular prevention
	Farnesyl transferase siRNA	Inhibits expression of farnesyl transferase	Improves pressure overload cardiac remodeling in mice [100]
	FPT-III	Farnesyl analog: inhibits farnesylation of Ras and other small GTPases	Cardioprotective effect in ischemia–reperfusion related to ischemic pre-conditioning [90]
	FTI-276	Tetrapeptid-mimetic, which inhibits FPPS	Improved cardiac remodeling in spontaneously hypertensive rats [101]
Silencing-mRNA	si-RNA-H-Ras	Inhibits mRNA synthesis of H-Ras	Inhibits cardiomyocyte hypertrophy in vitro by phenylephrine [25]
Signal inhibitors	Cyclosporin A	Calcineurin inhibitor	Attenuates pressure overload cardiac hypertrophy but causes HF [106]
	Rapamycin	Akt inhibitor	Prevented cardiac hypertrophy in a transgenic mouse model with SHP-2 active mutation [107]
	PD0325901	MEK inhibitor	Tested in cancer patients [111]
	SB203580	p38 inhibitor	Only tested in cancer cells

Examples of molecules used to inhibit Ras and its pathways in the heart, experimentally in animal models or clinically in patients. To our knowledge, the anti-cancer molecules PD0325901 and SB203580 have not yet been used in cardiac diseases, but are of interest as inhibitors of Ras-related pathways (more details in paragraph 6 of the article)

#### Farnesyl transferase inhibitors (FTIs)

These are a group of experimental anti-cancer drugs that block the farnesyl pyrophosphate synthase (FPPS), preventing the prenylation of Ras. FPPS inhibition in a transgenic mouse with a small interfering RNA (siRNA) of FPPS, resulted in inhibition of Ras activity and signaling pathway, with favorable effect on pressure overload-induced cardiac remodeling [100]. Also, the farnesyl transferase inhibitor, FTI-276, improved cardiac remodeling in spontaneously hypertensive rats [101].

### Ras modulation by mRNA modulators

A silencing inhibitor of RNA (siRNA) specific for H-Ras, si-H-Ras, inhibited rat cardiomyocytes hypertrophy in vitro [25]. miR-378, an endogenous microRNA and negative regulator of cardiac hypertrophy, blocked cardiomyocyte hypertrophy, as well as phosphorylation of Akt and ERK1/2, by knocking down the expression of Grb2 [102], upstream to Ras (Fig. 1).

### Antibodies against Ras

Recent experiments have shown that systemic administration of antibodies against Ras mutants exerted anti-tumor effects [103]. The antibodies against Ras used in experimental cancer therapy have a potential therapeutic effect in cardiovascular diseases, with high specificity and low toxicity [104].

### Regulation of other genes related to Ras

Newly discovered molecules in the Ras pathways have potential therapeutic applications. Carabin, a Ras-GAP protein and negative regulator of pro-hypertrophic signaling molecules calcineurin and Ras, is a potential therapeutic target in cardiac hypertrophy and HF [45]. Another modulator of the Ras pathway is the Raf kinase inhibitor protein (RKIP), which allows a more beneficial activation of the β-adrenergic pathway, avoiding long-term adverse effects of β-adrenergic stimulation, such as arrythmias and ventricular remodeling [105].

### Regulation of Ras pathways with small molecules

Other drugs have been used to counteract cardiac hypertrophy. For example, the immunosupresor Cyclosporine is a well-known inhibitor of calcineurin, and attenuates pressure-overload cardiac hypertrophy; however, it also causes HF, thus limiting its clinical use in that setting [106]. The macrolid Rapamycin, an immunosuppressant used in transplant, inhibits JNK and the Akt/mTOR complex, and prevented or even reversed cardiac hypertrophy in a transgenic mouse model with Ptpn11-Y279C that causes cardiac hypertrophy (Ptpn11 is a SHP2 protein, Fig. 1) [107]. Clinical trials in cancer patients with PDO325901, a specific MEK inhibitor, were terminated early due to ophthalmologic and neurologic toxicity [108].

## Conclusions and future directions

The central role of Ras in pathologic and physiologic cardiac hypertrophy has been demonstrated in multiple in vitro and in vivo settings. Recent studies showing cardio-protective effects of H-Ras modulation [25, 43, 88, 100, 101] open new therapeutic perspectives for pathological cardiac hypertrophy and HF.

Many questions remain, however, that deserve further studies. What specific pathways are activated by K-Ras and H-Ras so that one leads to hyperplasia, while the other leads to hypertrophy? Is there a role for Ras modulation in established HF, outside of pathological hypertrophy leading to HF? In addition, many obstacles remain before specific clinical applications of Ras modulation can be envisioned in cardiology. The use of a transgene of Ras in patients carries the risk of activating oncogenes. Another challenge is the lack of specificity of the FTI to pathological cardiac hypertrophy.

Despite more than three decades of research, no effective pharmacologic inhibitor of Ras has reached the clinical arena. However, recent data from studies on cells and animal models are highly promising. The better appreciation of the complexities of the Ras signaling network, as well as technological advances, have renewed the enthusiasm for therapies targeting Ras in cardiac pathologies.

## Data Availability

Not applicable.

## Abbreviations

Akt: Protein Kinase B (PKB)
AngII: Angiotensin II
ANF: Atrial Natriuretic Factor
AR: Adrenergic receptor
AT1R: Angiotensin-II receptor type 1
α1-AR: α1-Adrenergic receptor
β-AR: β-Adrenergic receptor
β-MHC: β-Myosin heavy chain
Ca^++^: Calcium
CaMKII: Ca^++^/calmodulin-dependent protein Kinase II
cAMP: Cyclic adenosine monophosphate
DAG: Diacylglycerol
DN-Ras: Dominant Negative Ras
eNOS: Endothelial nitric oxide synthase
Epac: Exchange protein directly activated by cAMP
ERK: Extracellular signal-regulated protein kinase
GAP: GTPase-activating protein
GDP: Guanosine Diphosphate
GEF: Guanine nucleotide exchange factor
GTP: Guanosine Triphosphate
G proteins: GTP-binding proteins
GPCR: G-protein coupled receptor
GSK: Glycogen Synthase Kinase
HCM: Hypertrophic cardiomyopathy
HF: Heart failure
HSG: Hyperplasia Suppressor Gene
IP_3_: Inositol trisphosphate
IP_3_R: Inositol trisphosphate receptor
JNK: Jun NH_2_-terminal kinase
LEOPARD: Lentigines, Electrocardiographic conduction defects, Ocular hypertelorism, Pulmonary stenosis, Abnormalities of the genitalia, Retarded growth, Deafness
LTCC: L-type Ca^++^ channel
MAPK: Mitogen-Activated Protein Kinase
MEK: MAPKK (MAP kinase kinase) that activates a MAPK (ERK)
NF1: Neurofibromin 1
NFAT: Nuclear Factor of Activated T cells
NF-kB: Nuclear Factor kappa B
PE: Phenylephrine
PDE: Phosphodiesterase
PI3K: Phosphoinositide 3-kinase
PIP2: Phosphatidylinositol 4,5-bisphosphate
PKA: Protein Kinase A
PKC: Protein Kinase C
PLC: Phospholipase C
PLN: Phospholamban
Ras: Rat sarcoma
Ras-opathies: Genetic diseases associated with mutations in the Ras pathway
RTK: Tyrosine Kinase receptor
RyR: Ryanodine receptor
SAPK: Stress Activated Protein Kinases
SEK-1: SAPK/ERK kinase
SERCA: Sarcoplasmic reticulum Ca^++^ ATPase
SkM- α Actin: Skeletal Muscle α Actin
Sos: Son of sevenless
TNF: Tumor Necrosis Factor
